# The effect of *m*Health-based exercise on *I*nsulin *S*ensitivity for patients with *H*epatocellular carcinoma and insulin resistance (mISH): protocol of a randomized controlled trial

**DOI:** 10.1186/s13063-022-06858-w

**Published:** 2022-11-08

**Authors:** Seung Mi Yeo, Joo Hyun Oh, Hee Ju Yu, Dong Hyun Sinn, Ji Hye Hwang

**Affiliations:** 1 Department of Rehabilitation Medicine, Pusan National University Yangsan Hospital, Pusan National University School of Medicine, Yangsan, Republic of Korea; 2 Department of Medicine, Nowon Eulji Medical Center, Eulji University School of Medicine, Seoul, Republic of Korea; 3 Department of Physical Therapy, Graduate School, Sahmyook University, Seoul, Republic of Korea; 4 Department of Medicine, Samsung Medical Center, Sungkyunkwan University School of Medicine, 81 Irwon-Ro Gangnam-Gu, Seoul, 06351 Republic of Korea; 5 Department of Physical and Rehabilitation Medicine, Samsung Medical Center, Sungkyunkwan University School of Medicine, 81 Irwon-Ro Gangnam-Gu, Seoul, 06351 Republic of Korea

**Keywords:** Hepatocellular carcinoma, mHealth, Insulin resistance, Exercise in cancer

## Abstract

**Background:**

The importance of insulin resistance is gaining increasing attention as it plays an important role in carcinogenesis in hepatocellular carcinoma (HCC). Although exercise is the most important intervention for lowering insulin resistance, it is not easy for HCC patients to maintain high compliance and do appropriate exercise. Mobile health (mHealth) with wearable devices can be the solution to carry out an adjusted and supervised exercise that can normalize insulin resistance in patients with HCC. We developed an HCC-specific application equipped with patient-centered exercise. In this paper, we present a randomized controlled trial protocol comparing an intervention group with a control group to determine whether mHealth-based exercise is effective in normalizing insulin sensitivity in HCC patients with insulin resistance after anticancer treatment.

**Methods:**

An assessor unblinded open label randomized controlled trial (RCT) will be conducted for 80 participants with treatment-naïve or recurrent HCC who have received treatment and achieved complete response at the time of screening. They will be randomly assigned (1:1) to one of two groups: an intervention group (*n* = 40) and a control group (*n* = 40). The intervention group will carry out mHealth-based exercise for 6 months from baseline, whereas the control group will receive the usual follow-up care for the first 3 months and mHealth-based exercise for the next 3 months. Both groups will be assessed at baseline, 3 months, and 6 months from baseline. The primary outcome is the normalized rate of insulin resistance in each group at 3 months. Insulin resistance is estimated by calculating homeostatic model assessment for insulin resistance (HOMA-IR). The secondary outcomes are body composition, physical fitness level, physical activity, and quality of life at 3 months.

**Discussion:**

This study is the first RCT to investigate the effect of mHealth-based home exercise with a wrist-wearable device on insulin sensitivity, physical fitness, and quality of life for HCC patients with insulin resistance. The result of this RCT will confirm not only safety and functional improvement but also biological effect when exercising using mHealth in HCC patients.

**Trial registration:**

ClinicalTrials.gov NCT04649671. Registered on 2 December 2020. The World Health Organization Trial Registration Data Set is not registered.

**Supplementary Information:**

The online version contains supplementary material available at 10.1186/s13063-022-06858-w.

## Introduction

The growing interest in the association between exercise and cancer has led to an increase in research on this topic. It is proven that exercise has a preventive effect in seven types of cancer (colon, breast, endometrial, kidney, bladder, esophagus, and stomach) and improves survival in three types of cancer (breast, colon and prostate) [1, 2]. In addition, there is sufficient evidence that exercise improves common cancer-related health outcomes in a variety of cancers, including anxiety, depressive symptoms, fatigue, physical functioning, and health-related quality of life [3]. However, little is known about the role of exercise for patients with hepatocellular carcinoma (HCC), which has the 4th highest mortality rate of all cancers [4].

Viral hepatitis in the East and alcohol or fatty liver/metabolic disease in the West are major risk factors for HCC. Fatty liver and metabolic diseases are currently changing as the major risk factors for HCC as westernization progresses and are expected to increase gradually [5]. Insulin resistance is one of the main mechanisms by which fatty liver and metabolic diseases cause HCC, and several studies have found a link between insulin resistance and HCC independent of viral hepatitis [6]. Kim et al. revealed that the incidence of HCC in patients with insulin resistance was significantly higher in multivariate analysis with hazard ratio of 3.24 (95% confidential interval 1.13–9.31) compared to patients without insulin resistance [7]. Similarly, Imai el al. also confirmed that the risk of HCC recurrence was significantly higher in patients with insulin resistance compared to patients without insulin resistance in multivariate analysis, with a hazard ratio of 1.48 (95% confidential interval 1.03–2.13) [8]. Therefore, it is necessary to treat insulin resistance in patients with a previous history of HCC, and the most basic and essential treatment for insulin resistance is exercise and diet [9]. In fact, animal studies have demonstrated the positive influence of moderate physical activity on HCC growth and progression [10, 11]. And a prospective cohort study suggested an inverse association between physical activity and the risk of HCC [12].

Exercise is expected to have a positive effect on patients with HCC, but patients and doctors consider hepatic decompensation to be a risk when exercising [13]. Only a few studies have reported the positive effects of in-hospital exercise for HCC patients [14–16]. Longer-term exercise interventions are necessary, especially for cancer patients, but it is not practical for all interventions to be carried in hospitals. Digital health, especially mobile health (mHealth) with wearable devices, is a promising solution that can allow patients with HCC to carry out adjusted and supervised exercise. The usefulness and effectiveness of mHealth has been reported for several cancers, such as breast [17] and prostate [18] cancer. Our previous study presented the efficacy and safety of the mHealth application and wearable devices, and their physical performance for patients with HCC. Our surveillance system, consisting of wearable Internet of Things (IoT) devices and HCC specific application, showed no complications or biochemical deterioration and showed an improved physical performance during the 12-week intervention period [19]. However, the biological improvement of insulin resistance was not confirmed, there was no control group for comparison, and the exercise intensity was manually readjusted once. Hence, we modified the HCC-specific application with monitored patient-centered exercises, and we planned a randomized controlled trial (RCT) using this application. The intervention group will carry out with mHealth-based exercises for 6 months and the control group will receive usual follow-up care for the first 3 months and mHealth-based exercise for the following 3 months.

Our hypothesis is that insulin resistance will be more significantly normalized, and physical fitness and quality of life (QoL) will be improved more significantly in the intervention group using the mobile application and exercise program for HCC patients than in the control group. This paper presents an open label 1:1 randomized controlled trial protocol comparing intervention and control groups to determine the effect of *m*Health-based exercise on *I*nsulin *S*ensitivity for patients with *H*CC and insulin resistance (mISH) after anticancer therapy.

## Methods/design

### Study design

This protocol is a two-arm, open-label RCT that compares usual follow-up care with mISH from baseline to 3 months. Participants will be collected in one academic hospital (Samsung Medical Center, South Korea).

### Study procedure

**Fig. 1:**
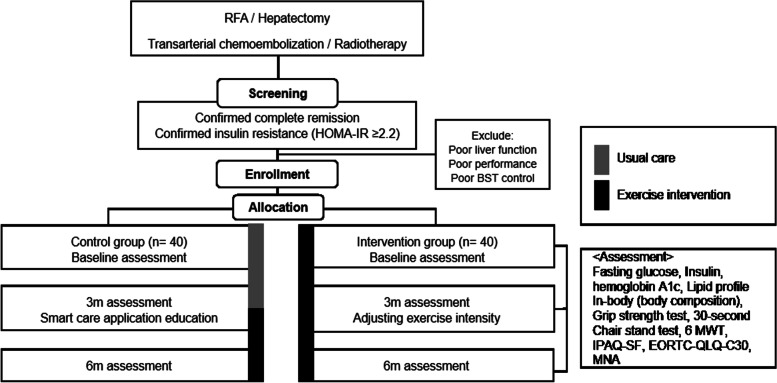
Participant flow through the randomized controlled trial. RFA, radiofrequency ablation; HOMA-IR, homeostatic model assessment for insulin resistance; 6 MWT, 6-min walk test; IPAQ-SF, Korean version international physical activity questionnaire-short form; EORTC-QOL-C30, European organization for research and treatment of cancer quality of life questionnaire C30; MNA, Mini nutritional assessment

Figure 1 outlines the flowchart of the RCT and Table 1 indicates the summary of baseline screening, enrollment, and assessment during study visits. The patients with treatment-naïve and recurrent HCC who have received treatment and achieved complete response at the time of screening will be screened about general condition, alcohol intake history, liver function, medical history, and drug history. A written consent including collection and use of participant data and biological specimens will be obtained from the participant by physician investigator.

**Table 1:**
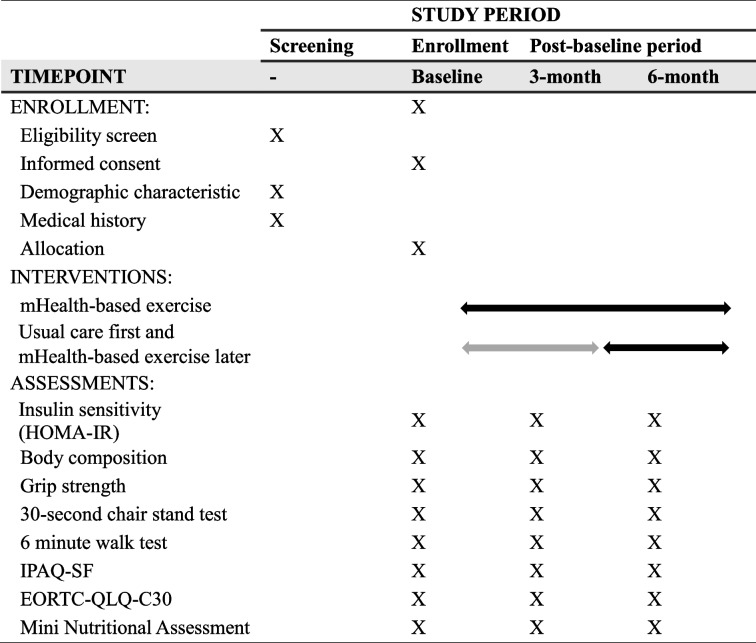
Summary of baseline screening, enrollment, and assessment during study visits

*mHealth* mobile health, *IPAQ-SF* International Physical Activity Questionnaire-Short Form, *EORTC-QLQ-C30* European Organization for Research and Treatment of Cancer-Quality of Life Questionnaire

The consent form states whether the participant consents to the use of data or if he or she chooses to withdraw from the trial. Because information obtained from blood tests is included in the outcomes of this study, consent for human material is also obtained. Informed consent materials are available from the corresponding author on request.

Homeostatic model assessment for insulin resistance (HOMA-IR) will be assessed in those who consented. Among screened participants, only patients with insulin resistance (HOMA-IR ≥ 2.2) will be enrolled in outpatient clinic. After enrollment, they need to be randomized based on 1:1 allocation to two groups: the intervention group should carry out mISH for 6 months, and the control group should receive the usual care for the first 3 months and mISH for the next 3 months. Participants should continue to take medications for other conditions as normal. In addition, the individual’s additional physical activity is not restricted. In both groups, assessments for the primary and secondary outcomes will be performed after 3 and 6 months from baseline.

After 3 months of usual care, 3 months of intervention was provided to the control group to avoid ethical issues. The randomized controlled trial design in this study is limited to comparing the outcomes at 3 months of the intervention group and the control group. The reason for planning the assessment of the outcomes at 6 months is to compare the outcomes at baseline, 3 months, and 6 months within the intervention group, and to compare the period of usual care with the period of intervention within the control group.

### Allocation, randomization, and blinding

Participants will be randomly assigned to the intervention group (*n* = 40), receiving 12 weeks of mISH or to the control group (*n* = 40), not receiving mISH, in a 1:1 ratio. Block randomization with an allocation ratio of 1:1 is implemented. The baseline assessments will be performed after allocation and randomization. During the study, the assessor has no choice but to see the participant wearing the wearable device (Dofit), and an open-label study will be conducted with assessors not being blinded.

### Participants

A total of 80 participants will be included in the study, 40 each in the intervention and control groups.

#### Inclusion criteria

- Adults aged 20–70
- Early-stage HCC, defined by modified International Union Against Cancer (UICC) stage 1 or 2
- Participants with treatment-naïve and recurrent HCC who have received treatment and achieved complete response at the time of screening
- Insulin resistance (homeostatic model assessment for insulin resistance (HOMA-IR) ≥ 2.2)
- Eastern Cooperative Oncology Group (ECOG) performance status of 0–2

#### Exclusion criteria

- Participants who disagree with the guidelines of the study
- Decreased liver function (Child Pugh class B or C)
- Bad general condition (Eastern cooperative oncology group (ECOG) > 2)
- Excessive drinkers (more than 20 g of alcohol intake per day)
- History of decompensated cirrhosis
- Inability to exercise due to severe heart and lung diseases (heart failure, ischemic heart disease, 3rd degree atrioventricular block, chronic obstructive pulmonary disease, severe high blood pressure (> 200/100 mmHg))
- Inability to exercise due to serious mental illness
- Use of insulin sensitizers (sulfonylurea, biguanide, thiazolidinediones, glucagon-like peptide-1 agonist, dipeptidyl peptidase-4 inhibitor) or those who take insulin
- Uncontrolled diabetes (hemoglobin A1c > 10%)

#### Criteria for discontinuing interventions

- If participants meet the exclusion criteria
- Tumor recurrence
- Participants request

### Intervention

A wearable IoT device and a mobile application-equipped exercise program specialized for HCC will be provided. The intervention group will use mISH for 6 months, whereas the control group will receive usual follow-up care for the first 3 months and mISH for the next 3 months. Personal exercise will be permitted during the trial.

### Intervention group

**Fig. 2:**
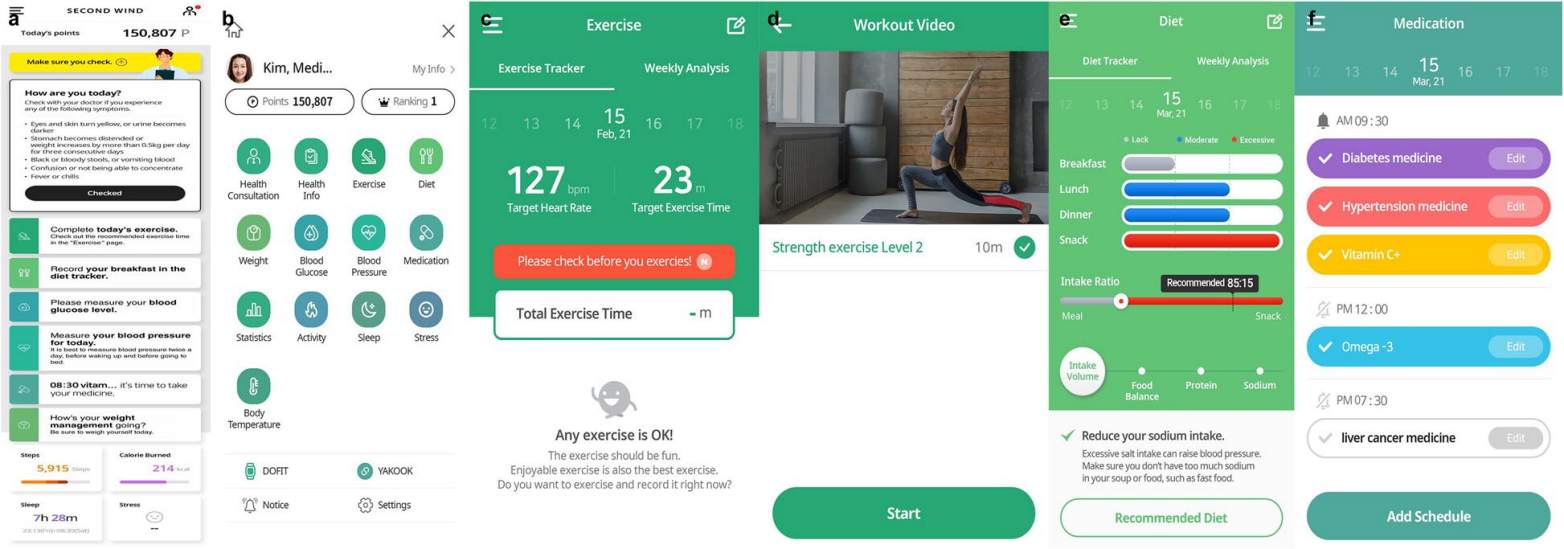
Application screenshots. Screenshots of the main page (**a**), menu tab (**b**), exercise management [aerobic exercise (**c**) and resistance exercise (**d**)], diet management (**e**), and medication management (**f**)

The mobile application, exercise program, and wearable IoT device comprise one intervention. The application uses data input from a wearable IoT device, Dofit (NF-B20, Medi Plus Solution, Seoul, South Korea). Dofit measures heart rate and physical activity (step counts and calories burned) when worn on the wrist. The application provides information on exercise management (aerobic and resistance exercises), activity tracking, diet management, medication management, and health consultation. Screenshots for each domain of the application are shown in Fig. 2. Each domain consists of the following: main page, menu tab, exercise management, activity tracking, diet management, medication management, and health consultation. Researchers will monitor and feedback on exercise performance.

#### Exercise management

The application offers aerobic and resistance exercises. For aerobic exercise, when the start button is pressed, the exercise time is recorded and the heart rate during exercise is measured and displayed in real-time. The intensity of aerobic exercises is prescribed at the target heart rate (THR) according to American College of Sports Medicine (ACSM) guidelines [20].

If a participant is enrolled less than 16 weeks after surgery or radiofrequency ablation (RFA), the intensity of aerobic exercise and resistance exercise is designed to increase step by step from week 1 to week 15. From week 1 to week 15, the intensity of aerobic exercise is set to increase from 58 to 68% of maximum heart rate and the intensity of resistance exercise is also set to increase (see Additional file 1). From the week 16 after surgery or RFA, general exercise management begins, and exercise intensity is adjusted according to the RPE.

If a participant is enrolled 16 weeks after surgery or RFA, baseline 6-min walk test (6 MWT) is used to control the initial intensity of exercise. If the baseline 6 MWT result is better than the average level for the same age and sex, the THR for initial aerobic exercise is 72% of the maximum heart rate, and general exercise management begins immediately. If the baseline 6 MWT result is less than the average level for the same age and sex, after maintaining the THR at 68% of maximum heart rate for 1 month, general exercise management begins. The average value of 6 MWT according to age and sex will be calculated using the following formula [21].

$$\mathrm{Men }\left(m\right) = \left(7.57 \times \mathrm{ height cm}\right)- \left(5.02 \times \mathrm{ age}\right)- \left(1.76 \times \mathrm{ weight kg}\right)- 309$$

$$\mathrm{Women }\left(m\right)= \left(2.11 \times \mathrm{ height cm}\right)- \left(2.29 \times \mathrm{ weight kg}\right)- \left(5.78 \times \mathrm{ age}\right)+ 667$$

In general exercise management, the THR of the next exercise is determined based on the RPE after the end of the previous exercise. If the participant records “very hard” even once, the exercise intensity is decreased, and if the participant records “very light” or “moderate” twice or more, the exercise intensity is increased. Exercise intensity is estimated by a RPE of 13 (a bit hard) to 15 (hard). In patients with hypertension, the THR is set lower. Participants can check their smart band to monitor their heart rate during aerobic exercise. Notifications are sent to them to inform them if the THR is achieved. This is useful to evaluate the participants’ compliance and to monitor if the participants exercise properly by looking for an increase in heart rate.

When general exercise management is started, the intensity of resistance exercise is also adjusted based on RPE and participants need to follow the video clips of exercises. Video exercise starts with a warm-up, resistance exercises using an elastic band, and finishes with stretching (cool-down).

Healthcare professionals and researchers will access exercise logs through a monitoring platform and will provide weekly guidance and positive reinforcement.

#### Activity tracking

Participants can review daily step counts using Dofit. The application displays daily step counts of general walking, fast walking, and running. It calculates and displays the calories burned from physical activity during the day.

#### Diet management

Based on the user’s body mass index, the necessary calories and customized daily diet guides specialized for HCC are provided. When participants enter the type and amount of food they eat, the application displays if the amount of carbohydrates and protein consumed is over or under the expert’s recommendation. Participants can also check if the balance of the food groups they eat is adequate.

#### Medication management

When medications are registered on the application, an alarm for timely medication is provided on the main screen.

#### Health consultation

The participants are informed of precautions necessary and complications that may occur depending on their cancer treatment process, and the app warns of symptoms that require a hospital visit. When participants have questions about their health or exercise, they can consult with physical therapists and physicians.

### Control group

All participants will receive the same brochure previously provided to HCC patients after anticancer therapy (radiofrequency ablation, hepatectomy, transarterial chemoembolization or radiotherapy). It lists guidelines for healthy living post anticancer therapy and diet specifications such as eating a high-protein diet, eating snacks in the late evening, and avoiding very spicy or salty foods and herbal medicines. An acceptable level of exercise intensity is also recommended in the brochure.

### Measurements

Data from electronic medical record will be used. All participants need to answer a self-administered questionnaire that includes a history of hypertension, diabetes, stroke, use of medications, alcohol, and smoking. Height and waist will be measured to the nearest half centimeter. Weight will be measured to the nearest half kilogram. Blood pressure will be measured after at least 10-min resting. The weight and waist will be measured at each assessment point.

Serum blood tests at baseline include the following: platelet count, hemoglobin A1c, fasting blood glucose, insulin, aspartate aminotransferase (AST), alanine aminotransferase (ALT), prothrombin time-international normalized ratio (PT INR), albumin, total bilirubin, alpha-fetoprotein, proteins induced by vitamin K absence of antagonist-II (PIVKA –II), high density lipoprotein cholesterol (HDL), triglyceride, and low density lipoprotein cholesterol (LDL). Among the above items, fasting glucose, insulin, hemoglobin A1c, and lipid profile will be measured at each assessment point.

### Assessments and data collection methods

Assessments, such as blood test (fasting glucose, insulin, hemoglobin A1c and lipid profile), physical activity, and QoL questionnaires, and physical fitness assessments will be carried out at baseline and 3 and 6 months after baseline. A nutritional assessment will be done to determine if there are any nutritional differences between the two groups. Table 1 describes the assessments for each time point.

#### Primary outcome

The primary outcome is the normalized rate of insulin resistance in each group at 3 months. Insulin resistance is estimated by calculating HOMA-IR, which uses the computer-based solution of the model provided by the Diabetes Trials Unit, Oxford Center for Diabetes, Endocrinology, and Metabolism (http://www.dtu.ox.ac.uk/homa). When the HOMA-IR falls below 2.2, it is considered that insulin resistance is normalized.

#### Secondary outcome

The secondary outcomes are the normalized blood test profile (fasting glucose, insulin, hemoglobin A1c), body composition, physical fitness, and patient-reported outcome such as physical activity level, QoL, and adverse events. Data for the body composition analyses is collected using a bioelectric impedance device, Inbody 720 (Biospace). Muscle mass and body fat percentage will be recorded, and body mass index (BMI) will be calculated as body weight/height^2^ (kg/m^2^).

Physical fitness is measured by the grip strength test, the 30-s chair stand test, and the 6 MWT. A digital hand-held dynamometer (microFET® HandGRIP; Hoggan Health, Salt Lake City, UT) is used to assess the upper extremity muscle strength. Seated participants hold the dynamometer in their arm with shoulder adducted, elbow flexed to 90°, and forearm and wrists resting in a neutral position. After 3 s, the maximum power is measured. The experiment is performed on the other side in the similar way. We use an average of the 3 grip strength tests. A 30-s chair stand test is performed to evaluate the strength of the lower extremities. Participants are made to sit straight in a chair without the support of the backrest and with both arms folded across the chest. For 30 s, they complete stand-up and sit-down motions as quickly as possible, and the number of motions is counted. To estimate the level of cardiopulmonary endurance, the 6 MWT is conducted. The total distance walked at maximal speed for 6 min is recorded.

Physical activity levels are objectively measured using the International Physical Activity Questionnaire-Short Form (IPAQ-SF) questionnaire. This questionnaire includes 9 questions on time spent on vigorous and moderate activities and time spent walking or sitting for the preceding 7 days. The total number of metabolic equivalents (METs) per week can be calculated using this questionnaire. The calculated physical activity is classified into 3 IPAQ-SF categories: inactive (< 600 METs), minimally active (≥ 600 to < 3000), and highly active (≥ 3000).

General health-related QoL is assessed by the European Organization for Research and Treatment of Cancer Quality of Life Questionnaire C30 (EORTC-QLQ-C30). The questionnaire includes 30 questions regarding global health status, functional scales, and symptom scales. Higher scores on the general health status and functional scales indicate positive outcomes, while high symptom scales are considered negative. Adverse events are also reported using EORTC-QLQ-C30.

General nutritional status is assessed by the mini nutritional assessment (MNA). The MNA consists of 18 questions derived from four parameters of assessment: anthropometric, general, dietary, and subjective. The total score for the full MNA will fall between 0 and 30 points: 24 and higher indicates a well-nourished patient; 17 to 23.5 indicates a risk of malnutrition; lower than 17 indicates malnutrition.

#### Plans to promote participant retention and complete follow-up

In this study, the control group will be given mHealth after 3 months of usual care, a benefit that may reduce the dropout of the control group. In addition, wearable devices will be provided free of charge so that they could be used even after the end of the study. To assist the participants, we will provide 24-h telephone number in the event of a problem and the participants who discontinue or deviate from the protocol will be encouraged to undergo assessments to implement the intention-to-treat data analysis at the end of the study.

### Data management

Data is entered in the order in which it is collected. Assessment results are recorded on a paper-based Case Report Form (CRF), and the data is coded in computer on the day of the assessment. All the participant data is coded by research team members and is stored and password protected on a secured platform accessible only by senior investigators. Backup database will be updated regularly. Paper-based CRFs will be kept in a secure locker for 10 years after the end of the study in accordance with the guidelines for storing research-related documents. The anonymized dataset will be available on request to the corresponding authors. No interim analysis will be performed prior to the end of the study.

### Sample size calculation

The target number of participants was calculated using the nQuery 8.5 program with the guidance of biostatisticians of Samsung Medical Center. Assuming that the normalized rate of HOMA-IR in the intervention group is 30% and 5% in the control group [22], 36 participants per group are required to achieve 80% power and 5% significance level. Considering a 10% dropout rate, a final 40 participants need to be recruited for each group.

### Statistical analysis

The strategy for statistical analysis is developed under the supervision of a biostatistician at the Samsung Medical Center. Analyses will be performed using an intention-to-treat approach. Descriptive statistics will be used to present baseline characteristics of both groups. For the primary outcome, the proportion of participants in each group with normalized HOMA-IR at 3 months after baseline will be compared using the chi-square test. For the secondary outcomes, changes in blood test (fasting glucose, insulin, hemoglobin A1c and lipid profile), body composition (muscle mass, body fat), physical fitness level (grip strength test, 30 s chair stand test, 6 MWT), physical activity (IPAQ-SF), general health-related quality of life (EORTC-QLQ-C30), and adverse events at 3 months after baseline will be compared by an unpaired *t*-test. In the control group, according to the self-controlled study design, all outcomes after 3 months of usual care and after using mISH for 3 months will be compared by paired *t*-test. In the intervention group, the primary and secondary outcomes of baseline, 3 months, and 6 months of intervention will be analyzed with repeated measured ANOVA to check whether there are significant changes over time. All tests will use a 5% level of significance (*p* < 0.05). Relative risks and 95% confidence intervals are reported for primary outcome.

## Oversight and monitoring

### Composition of the coordinating center and trial steering committee

There is no coordinating center or study steering committee in this study. This study is conducted in a single center without funding. Gastroenterology research team who enrolls participants and Rehabilitation medicine research team is responsible for patient evaluation and intervention management meet regularly to evaluate the performance and progress of the study and ensure that the study protocol is followed. The entire research team, including the two principal investigators, meets every 3 months to discuss the management and financial issues related to the study. To ensure that the protocol is implemented as planned, the principle investigators are responsible for managing and overseeing the study and providing guidance and administrative support.

### Participant safety and withdrawal

The potential risk level reviewed by institutional review board and principal investigator is minimal. For prevention and management in adverse events, participants can call researchers at any time if they have any question or problems. If there are an intolerable muscle pain or injury, they will visit a hospital and will be examined their condition by principal investigator.

All participants are able to discontinue voluntarily the study at any time and they can be withdrawn in case of the significant disease non-related to study, and not following instruction of doctor in charge.

### Auditing

The need for an audit is not anticipated due to low-risk intervention. However, the study may be audited by the institutional review board (IRB) center of the hospital where the study will be conducted.

## Ethics and dissemination

This study was approved by the IRB of Samsung Medical Center (number: 2020–06-037). Current protocol version is 2.3 and approved on 27 October 2021. In the event of important protocol modifications, it will be reported to the IRB and approved after the consent of the physician investigator and co-researchers. The trial was prospectively registered on the ClnicalTrials.gov website (trial number: NCT04649671). The dissemination of research will occur through several pathways. The research results will be submitted for publication to peer-reviewed journals, both nationally and internationally. The protocol adheres to the recommended Standard Protocol Items: Recommendations for Interventional Trials (SPIRIT) checklist (see Additional file 2). The datasets analyzed during the current study and statistical code are available from the corresponding author on reasonable request, as is the full protocol.

## Discussion

Insulin resistance is known as one of the main mechanisms by which fatty liver and metabolic diseases cause HCC, and there are several hypotheses about the mechanism by which insulin resistance causes HCC [23]. Hyperinsulinemia due to insulin resistance increases the production of the peptide hormone insulin-like growth factor (IGF-I), which stimulates cell growth through proliferation of cells and inhibition of apoptosis in the liver [24]. IGF-I activates the insulin receptor substrate 1 (IRS-1) protein, which is known to be up-regulated in human HCC, and induces malignant transformation through activation of the mitogen-activated protein kinase (MAPK) cascade [25]. There are reports that states lipotoxicity due to the accumulation of fat in the liver increases reactive oxygen, and DNA damage caused by oxidative stress increases the incidence of HCC [24, 26].

Treatment of insulin resistance in patients with a history of HCC can reduce the recurrence of HCC, and the most important treatment for insulin resistance is exercise and diet control. In fact, several studies have reported that physical activity is inversely correlated with the incidence of HCC [11, 12, 27]. However, guidelines for exercise and diet have not yet been established in HCC patients, and there are only a few reported prospective studies. Similarly, the positive effects of “in-hospital exercise” for patients with HCC have been reported in a few studies [14–16].

New exercise system can be realized using mHealth as it is possible to adjust the intensity of exercise based on real-time feedback and in-application chat with experts, and to monitor any dangerous symptoms. Moreover, the mHealth-based exercise can be performed anywhere, at any moment. Previously, we have confirmed that the application and exercise program using Dofit are safe and effective [19]. This RCT will be used as the upgraded application with exercise intensity control algorithm and intends to investigate the effectiveness of mHealth by comparison with a control group. The intervention group will work out with mHealth for 6 months, and the control group will receive usual care for the first 3 months and mHealth-based exercise for the next 3 months.

This study has several strengths: to the best of our knowledge, this is the first RCT to demonstrate the beneficial effect of mHealth-based exercise with wearable IoT device in HCC patients with insulin resistance. Second, this RCT investigates if there is a biological effect on insulin sensitivity, physical fitness, and QoL. Third, as participants are prescribed longer-term exercise interventions when compared to previous studies, this study can ensure adherence to long-term mHealth-based exercises.

One limitation of this study is that it is difficult to determine the generalizability to other HCC patients because only subjects with complete remission and preserved liver function participate in this study.

In conclusion, the results of this RCT can provide evidence for mHealth to be a safe way to enable exercising by patients with HCC.

### Trial status

ClinicalTrials.gov Identifier: NCT04649671. First posted on December 2, 2020; last update posted on February 25, 2022. Actual study started on March 16, 2021, and estimated study completion date is October 31, 2023. https://clinicaltrials.gov/ct2/show/NCT04649671

## Supplementary Information

**Additional file 1. **Exercise protocols for post-op rehabilitation in hepatocellular carcinoma patients.

**Additional file 2. **SPIRIT Checklist

## Data Availability

Study results will be published in peer-reviewed journals and presented at national and international conferences. Upon completion of the study, all relevant data will be shared by the corresponding author upon reasonable request.

## Abbreviations

HCC: Hepatocellular carcinoma
mHealth: Mobile health
IoT: Internet of Things
RCT: Randomized controlled trial
RPE: Rating of perceived exertion
QoL: Quality of life
mISH: MHealth-based exercise on Insulin Sensitivity for patients with HCC and insulin resistance
HOMA-IR: Homeostatic model assessment for insulin resistance
UICC: International Union Against Cancer
THR: Target heart rate
ACSM: American College of Sports Medicine
RFA: Radiofrequency ablation
6 MWT: 6-Minute walk test
AST: Aspartate aminotransferase
ALT: Alanine aminotransferase
PT INR: Prothrombin time-international normalized ratio
PIVKA –II: Proteins induced by vitamin K absence of antagonist-II
HDL: High density lipoprotein cholesterol
LDL: Low density lipoprotein cholesterol
BMI: Body mass index
IPAQ-SF: International Physical Activity Questionnaire-Short Form
METs: Metabolic equivalents
EORTC-QLQ-C30: European Organization for Research and Treatment of Cancer Quality of Life Questionnaire C30
MNA: Mini nutritional assessment
RMANOVA: Repeated measured analysis of variance analysis
IRB: Institutional review board
SPIRIT: Standard protocol items: recommendations for interventional trials
