# Supplementary material for: The effect of mHealth-based exercise on Insulin Sensitivity for patients with Hepatocellular carcinoma and insulin resistance (mISH): protocol of a randomized controlled trial
Source: Trials. 2022 Nov 8;23:930. doi: 10.1186/s13063-022-06858-w (PMC9641912; doi:10.1186/s13063-022-06858-w)
Supplement: Supplementary file 1 — Additional file 1. Exercise protocols for post-op rehabilitation in hepatocellular carcinoma patients. [file 13063_2022_6858_MOESM1_ESM.docx]

| **Exercise / Phase** | **Step 1 (post-op 2–3 weeks)** | ^*^**Step 2 (post-op 4–7 weeks) & Step 3 (post-op 8–15 weeks)** | **Step 4 (post-op 16 weeks)** |
| --- | --- | --- | --- |
| Warm up | 1. Shoulder rotation stretch | 1. Abdominal breathing | 1. Abdominal breathing |
|  | 2. Cross-body shoulder stretch | 2. Shoulder rotation stretch | 2. Shoulder rotation stretch |
|  | 3. Trunk rotation (left/right) | 3. Hip wings | 3. Hip wings |
|  | 4. Above-the-head chest stretch | 4. Knee circles | 4. Knee circles |
|  | 5. Hamstring stretch |  |  |
|  | 6. Stretching the back and leg |  |  |
|  | 7. Standing side stretch (left/right) |  |  |
|  | 8. Hip wings |  |  |
| Main exercise | 1. High knee | 1. Leg swings (front/side/back) | 1. Squat without chair |
|  | 2. Leg swings (front/side/back) | 2. Shake leg (left/right) | 2. Quadriceps stretch |
|  | 3. Knee circles | 3. Chair squat | 3. Hold for 30 seconds in the squat position |
|  | 4. Arm circles with 90° arm abduction | 4. Quadriceps stretch | 4. Quadriceps stretch |
|  | 5. Triceps stretch | 5. Scapular retraction with resistance in sitting | 5. Elbow flexion with resistance in standing |
|  | 6. Overhead arm stretch | 6. Shoulder rotation stretch | 6. Shoulder abduction with resistance in standing |
|  |  | 7. Elbow flexion with resistance in sitting | 7. Shoulder rotation stretch |
|  |  | 8. Shoulder abduction with resistance in sitting | 8. Shoulder extension with resistance in standing |
|  |  | 9. Cross-body shoulder stretch | 9. Triceps stretch |
| Cool down | 1. Shake wrist (left/right) | 1. Shake wrist (left/right) | 1. Knee circles |
|  | 2. Shake leg (left/right) | 2. Overhead arm stretch | 2. Shake leg (left/right) |
|  | 3. Abdominal breathing | 3. Abdominal breathing | 3. Shake wrist (left/right) |
|  |  |  | 4. Overhead arm stretch |
|  |  |  | 5. Abdominal breathing |

**Additional file 1**. Exercise protocols for post-op rehabilitation in hepatocellular carcinoma patients

^*^ Steps 2 and 3 have differences in the number of repetitions required.
