# Supplementary material for: The effect of mHealth-based exercise on Insulin Sensitivity for patients with Hepatocellular carcinoma and insulin resistance (mISH): protocol of a randomized controlled trial
Source: Trials. 2022 Nov 8;23:930. doi: 10.1186/s13063-022-06858-w (PMC9641912; doi:10.1186/s13063-022-06858-w)
Supplement: Supplementary file 2 — Additional file 2. SPIRIT Checklist [file 13063_2022_6858_MOESM2_ESM.docx]

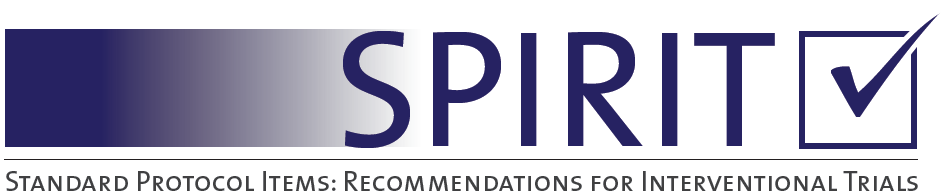


SPIRIT 2013 Checklist: Recommended items to address in a clinical trial protocol and related documents*

| Section/item | Item No | Description | Elaboration |
| --- | --- | --- | --- |
| **Administrative information** | | |  |
| Title | 1 | Descriptive title identifying the study design, population, interventions, and, if applicable, trial acronym | page 1, Title, paragraph 1-3 |
| Trial registration | 2a | Trial identifier and registry name. If not yet registered, name of intended registry | page 4, Trial registration number, paragraph 54-56 |
|  | 2b | All items from the World Health Organization Trial Registration Data Set | page 4, Trial registration number, paragraph 54-56 |
| Protocol version | 3 | Date and version identifier | page 18, Ethics and dissemination, paragraph 400 |
| Funding | 4 | Sources and types of financial, material, and other support | page 22, Funding, paragraph 488-491 |
| Roles and responsibilities | 5a | Names, affiliations, and roles of protocol contributors | page 1-2, paragraph 5-26, page 23, Author’s contributions, paragraph 498-501 |
|  | 5b | Name and contact information for the trial sponsor | page 22, Funding, paragraph 488-491 |
|  | 5c | Role of study sponsor and funders, if any, in study design; collection, management, analysis, and interpretation of data; writing of the report; and the decision to submit the report for publication, including whether they will have ultimate authority over any of these activities | page 22, Funding, paragraph 488-491 |
|  | 5d | Composition, roles, and responsibilities of the coordinating centre, steering committee, endpoint adjudication committee, data management team, and other individuals or groups overseeing the trial, if applicable (see Item 21a for data monitoring committee) | page 17, Oversight and monitoring, paragraph 372-382 |
| Introduction |  |  |  |
| Background and rationale | 6a | Description of research question and justification for undertaking the trial, including summary of relevant studies (published and unpublished) examining benefits and harms for each intervention | page 5-7, Introduction, paragraph 60-111 |
|  | 6b | Explanation for choice of comparators | page 6, Introduction, paragraph 98-102 |
| Objectives | 7 | Specific objectives or hypotheses | page 6-7, Introduction, paragraph 105-111 |
| Trial design | 8 | Description of trial design including type of trial (eg, parallel group, crossover, factorial, single group), allocation ratio, and framework (eg, superiority, equivalence, noninferiority, exploratory) | page 6-7, Introduction, paragraph 108-111 |
| Methods: Participants, interventions, and outcomes | | |  |
| Study setting | 9 | Description of study settings (eg, community clinic, academic hospital) and list of countries where data will be collected. Reference to where list of study sites can be obtained | page 7, Study design, paragraph 113-117 |
| Eligibility criteria | 10 | Inclusion and exclusion criteria for participants. If applicable, eligibility criteria for study centres and individuals who will perform the interventions (eg, surgeons, psychotherapists) | page 8-9, Participants, paragraph 149-173 |
| Interventions | 11a | Interventions for each group with sufficient detail to allow replication, including how and when they will be administered | page 9, Intervention, paragraph 180-184 |
|  | 11b | Criteria for discontinuing or modifying allocated interventions for a given trial participant (eg, drug dose change in response to harms, participant request, or improving/worsening disease) | page 18, Participant safety and withdrawal, paragraph 384-391 |
|  | 11c | Strategies to improve adherence to intervention protocols, and any procedures for monitoring adherence (eg, drug tablet return, laboratory tests) | page 10, Exercise management, paragraph 198-233 |
|  | 11d | Relevant concomitant care and interventions that are permitted or prohibited during the trial | page 7-8, Study procedure, paragraph 119-139 |
| Outcomes | 12 | Primary, secondary, and other outcomes, including the specific measurement variable (eg, systolic blood pressure), analysis metric (eg, change from baseline, final value, time to event), method of aggregation (eg, median, proportion), and time point for each outcome. Explanation of the clinical relevance of chosen efficacy and harm outcomes is strongly recommended | page 14, 15, 16 Primary outcome, Secondary outcome, paragraph 287-330 |
| Participant timeline | 13 | Time schedule of enrolment, interventions (including any run-ins and washouts), assessments, and visits for participants.  A schematic diagram is highly recommended (see Figure 1) | page 7-8, Study procedure, paragraph 119-139 |
| Sample size | 14 | Estimated number of participants needed to achieve study objectives and how it was determined, including clinical and statistical assumptions supporting any sample size calculations | page 16, Sample size calculation, paragraph 350-355 |
| Recruitment | 15 | Strategies for achieving adequate participant enrolment to reach target sample size | page 7, Study procedure, paragraph 119-130 |
| **Methods: Assignment of interventions (for controlled trials)** | | |  |
| Allocation: |  |  |  |
| Sequence generation | 16a | Method of generating the allocation sequence (eg, computer-generated random numbers), and list of any factors for stratification. To reduce predictability of a random sequence, details of any planned restriction (eg, blocking) should be provided in a separate document that is unavailable to those who enrol participants or assign interventions | page 8, Allocation, randomization, and blinding, paragraph 141-147 |
| Allocation concealment mechanism | 16b | Mechanism of implementing the allocation sequence (eg, central telephone; sequentially numbered, opaque, sealed envelopes), describing any steps to conceal the sequence until interventions are assigned | page 8, Allocation, randomization, and blinding, paragraph 141-147 |
| Implementation | 16c | Who will generate the allocation sequence, who will enrol participants, and who will assign participants to interventions | page 8, Allocation, randomization, and blinding, paragraph 141-147 |
| Blinding (masking) | 17a | Who will be blinded after assignment to interventions (eg, trial participants, care providers, outcome assessors, data analysts), and how | page 8, Allocation, randomization, and blinding, paragraph 141-147 |
|  | 17b | If blinded, circumstances under which unblinding is permissible, and procedure for revealing a participant’s allocated intervention during the trial | page 8, Allocation, randomization, and blinding, paragraph 141-147 |
| **Methods: Data collection, management, and analysis** | | |  |
| Data collection methods | 18a | Plans for assessment and collection of outcome, baseline, and other trial data, including any related processes to promote data quality (eg, duplicate measurements, training of assessors) and a description of study instruments (eg, questionnaires, laboratory tests) along with their reliability and validity, if known. Reference to where data collection forms can be found, if not in the protocol | page 14, 15, 16 Primary outcome, Secondary outcome, paragraph 287-330 |
|  | 18b | Plans to promote participant retention and complete follow-up, including list of any outcome data to be collected for participants who discontinue or deviate from intervention protocols | page 15, Plans to promote participant retention and complete follow-up, paragraph 332-338 |
| Data management | 19 | Plans for data entry, coding, security, and storage, including any related processes to promote data quality (eg, double data entry; range checks for data values). Reference to where details of data management procedures can be found, if not in the protocol | page 16, Data management, paragraph 340-348 |
| Statistical methods | 20a | Statistical methods for analysing primary and secondary outcomes. Reference to where other details of the statistical analysis plan can be found, if not in the protocol | page 16-17, Statistical analysis, paragraph 357-370 |
|  | 20b | Methods for any additional analyses (eg, subgroup and adjusted analyses) | page 17, Statistical analysis, paragraph 367-370 |
|  | 20c | Definition of analysis population relating to protocol non-adherence (eg, as randomised analysis), and any statistical methods to handle missing data (eg, multiple imputation) | page 16-17, Statistical analysis, paragraph 357-370 |
| **Methods: Monitoring** | | |  |
| Data monitoring | 21a | Composition of data monitoring committee (DMC); summary of its role and reporting structure; statement of whether it is independent from the sponsor and competing interests; and reference to where further details about its charter can be found, if not in the protocol. Alternatively, an explanation of why a DMC is not needed | page 18, Auditing, paragraph 393-396 |
|  | 21b | Description of any interim analyses and stopping guidelines, including who will have access to these interim results and make the final decision to terminate the trial | page 16, Data management, paragraph 340-348 |
| Harms | 22 | Plans for collecting, assessing, reporting, and managing solicited and spontaneously reported adverse events and other unintended effects of trial interventions or trial conduct | page 18, Participants safety and withdrawal, paragraph 384-391 |
| Auditing | 23 | Frequency and procedures for auditing trial conduct, if any, and whether the process will be independent from investigators and the sponsor | page 18, Auditing, paragraph 393-396 |
| Ethics and dissemination | | |  |
| Research ethics approval | 24 | Plans for seeking research ethics committee/institutional review board (REC/IRB) approval | page 18-19, Ethics and dissemination, paragraph 398-409 |
| Protocol amendments | 25 | Plans for communicating important protocol modifications (eg, changes to eligibility criteria, outcomes, analyses) to relevant parties (eg, investigators, REC/IRBs, trial participants, trial registries, journals, regulators) | page 18-19, Ethics and dissemination, paragraph 398-409 |
| Consent or assent | 26a | Who will obtain informed consent or assent from potential trial participants or authorised surrogates, and how (see Item 32) | page 7, Study procedure, paragraph 119-132, page 22, Ethics approval and consent to participate, paragraph 474-478 |
|  | 26b | Additional consent provisions for collection and use of participant data and biological specimens in ancillary studies, if applicable | page 7, Study procedure, paragraph 119-125 |
| Confidentiality | 27 | How personal information about potential and enrolled participants will be collected, shared, and maintained in order to protect confidentiality before, during, and after the trial | page 16, Data management, paragraph 340-348 |
| Declaration of interests | 28 | Financial and other competing interests for principal investigators for the overall trial and each study site | page 22, Competing interests, paragraph 485-486 |
| Access to data | 29 | Statement of who will have access to the final trial dataset, and disclosure of contractual agreements that limit such access for investigators | page 16, Data management, paragraph 340-348 |
| Ancillary and post-trial care | 30 | Provisions, if any, for ancillary and post-trial care, and for compensation to those who suffer harm from trial participation | _____N/A_____ |
| Dissemination policy | 31a | Plans for investigators and sponsor to communicate trial results to participants, healthcare professionals, the public, and other relevant groups (eg, via publication, reporting in results databases, or other data sharing arrangements), including any publication restrictions | page 18, Ethics and dissemination, paragraph 398-409 |
|  | 31b | Authorship eligibility guidelines and any intended use of professional writers | page 23, Author’s contributions, paragraph 498-501 |
|  | 31c | Plans, if any, for granting public access to the full protocol, participant-level dataset, and statistical code | page 18, Ethics and dissemination, paragraph 398-409 |
| Appendices |  |  |  |
| Informed consent materials | 32 | Model consent form and other related documentation given to participants and authorised surrogates | page 7, Study procedure, paragraph 127-130 |
| Biological specimens | 33 | Plans for collection, laboratory evaluation, and storage of biological specimens for genetic or molecular analysis in the current trial and for future use in ancillary studies, if applicable | _____N/A_____ |

*It is strongly recommended that this checklist be read in conjunction with the SPIRIT 2013 Explanation & Elaboration for important clarification on the items. Amendments to the protocol should be tracked and dated. The SPIRIT checklist is copyrighted by the SPIRIT Group under the Creative Commons “[Attribution-NonCommercial-NoDerivs 3.0 Unported](http://www.creativecommons.org/licenses/by-nc-nd/3.0/)” license.
